# Human milk banks in the response to COVID-19: a statement of the regional human milk bank network for Southeast Asia and beyond

**DOI:** 10.1186/s13006-021-00376-2

**Published:** 2021-03-29

**Authors:** Estrella Olonan-Jusi, Paul G. Zambrano, Vu H. Duong, Nguyen T. T. Anh, Nant S. S. Aye, Mei Chien Chua, Hikmah Kurniasari, Zaw Win Moe, Sopapan Ngerncham, Nguyen T. T. Phuong, Janice Datu-Sanguyo

**Affiliations:** 1 Human Milk Bank Association of the Philippines, Manila, Philippines; 2 Alive & Thrive Southeast Asia/FHI 360, 1101 Quezon City, Philippines; 3 Alive & Thrive Southeast Asia/FHI 360, Hanoi, Vietnam; 4 Human Milk Bank, Tu Du Hospital, Ho Chi Minh City, Vietnam; 5 Neonatal Intensive Care Unit, Central Women’s Hospital, Yangon, Myanmar; 6 Department of Neonatology and KK Human Milk Bank, KK Women’s and Children’s Hospital, Singapore, Singapore; 7 Human Milk Bank Initiative Association of Indonesia, Jakarta, Indonesia; 8 Neonatal Intensive Care Unit, Yankin Children’s Hospital, Yangon, Myanmar; 9 Siriraj Human Milk Bank, Department of Pediatrics, Faculty of Medicine Siriraj Hospital, Mahidol University, Bangkok, Thailand; 10 Human Milk Bank, Da Nang Hospital for Women and Children, Da Nang, Vietnam; 11 Alive & Thrive Southeast Asia/FHI 360, Manila, Philippines

**Keywords:** COVID-19, Human milk bank, Breastfeeding, Donor human milk, Pasteurization, Regional network

## Abstract

**Background:**

The World Health Organization (WHO) recommendations on infant feeding in the context of COVID-19 uphold standing recommendations for breastfeeding, non-separation, and skin-to-skin contact, including the use of donor human milk when mother’s own milk is not available.

**Insufficient guidance on the use of donor human milk and the role of human milk banks in the pandemic response:**

COVID-19 clinical management guidelines in seven countries in Southeast Asia are not aligned with WHO recommendations despite the lack of evidence of transmission through either breastmilk or breastfeeding. The use of safe donor human milk accessed through human milk banks is also insufficiently recommended, even in countries with an existing human milk bank, leading to a gap in evidence-based management of COVID-19. This highlights long-standing challenges as well as opportunities in the safe, equitable, and resilient implementation of human milk banks in the region.

**Conclusions:**

This statement reflects the expert opinion of the Regional Human Milk Bank Network for Southeast Asia and Beyond on the need to revisit national guidelines based on the best evidence for breastfeeding during the COVID-19 pandemic, to incorporate human milk bank services in national obstetric and newborn care guidelines for COVID-19 where possible, and to ensure that operations of human milk banks are adapted to meet the needs of the current pandemic and to sustain donor human milk supply in the long-term. The Network also recommends sustained engagement with the global human milk bank community.

**Supplementary Information:**

The online version contains supplementary material available at 10.1186/s13006-021-00376-2.

## Background

The COVID-19 pandemic has affected all ten member states of the Association of Southeast Asian Nations or ASEAN. All countries have taken measures to limit transmission of the virus, and for those with severe illness to receive appropriate, life-saving treatment. As the world learns more about the virus, and how best to respond to the threat, the need for evidence-based approaches in all aspects of the COVID-19 response remains paramount.

Even before the pandemic, global evidence on the role of breastfeeding in preventing maternal and child illness and death, and its long-term health and economic impact for children, mothers, and even nations is stronger than it has ever been [1, 2]. The COVID-19 pandemic has not diminished this evidence. While a number of case studies reported the detection of SARS-CoV-2 RNA in breastmilk samples of women who tested positive for COVID-19 [3–5], more robust research suggests that breastmilk is not a route of vertical transmission [6]. Antibodies for SARS-CoV-2 have even been detected in breastmilk, suggesting a potential protective benefit [7, 8].

The World Health Organization (WHO) recommends that mothers with suspected or confirmed COVID-19 should not be separated from their infants, and breastfeeding recommendations practiced with additional infection prevention and control measures such as mask wearing, hand hygiene and wiping of surfaces [9]. Health facilities and staff are also advised to support mothers to initiate breastfeeding within one hour of birth, practice skin-to-skin contact, to share a room, and to continue to breastfeed safely [10]. WHO advises that newborn care guidelines must be based on a full consideration of not only the potential risk of COVID-19 infection of the infant, but also the risks of morbidity and mortality associated with not breastfeeding, the deprivation of the protective effects of skin-to-skin contact, and the risks associated with breastmilk substitutes [11].

## Insufficient guidance on the use of donor human milk and the role of human milk banks in the pandemic response

### Inconsistencies in country guidelines

Despite the clear recommendations and supporting evidence, governmental and professional medical association guidelines on obstetric and newborn care for COVID-19 in countries in Southeast Asia were inconsistent with WHO guidance. A review of guidelines on pregnancy, intrapartum and postpartum care in the context of COVID-19 from 33 countries, including seven in Southeast Asia, collected from 21 March and 30 April 2020, found that Indonesia, Malaysia, the Philippines, and Singapore did not recommend skin-to-skin contact, early initiation of breastfeeding and rooming-in for mothers with confirmed COVID-19 [12]. Thailand also did not recommend skin-to-skin contact and early initiation but allowed rooming-in with two meters distance between the mother and the infant based on family’s preference [12]. There was no mention of skin-to-skin contact and early initiation of breastfeeding in the Myanmar and Vietnam guidelines, but Myanmar recommended unrestricted rooming-in while Vietnam recommended immediate separation [12]. Specific to infant feeding, Indonesia recommended direct breastfeeding with infection protection and control measures, Myanmar and the Philippines allowed breastfeeding based on family preference, while Malaysia, Singapore, Thailand, and Vietnam did not recommend it [12]. If direct breastfeeding is not possible, Myanmar, Philippines and Vietnam recommended the provision of expressed breastmilk [12]. It is also noteworthy that only the Vietnam guidelines explicitly recommended the provision of donor human milk when indicated [12].

Some of the countries later updated their guidelines or issued related guidelines to align with the WHO recommendations. The Thailand guidelines for care of newborns who were born from mothers with suspected or confirmed COVID-19 issued in April 2020 and the Philippines guidelines for the management of women about to give birth and newborns issued in July 2020 allowed skin-to-skin contact, early initiation of breastfeeding and direct breastfeeding under strict droplets precaution based on the mother’s preference after proper counselling [13, 14]. The KK Women’s and Children’s Hospital in Singapore, one of the largest hospitals in the country, issued its own guidance for breastfeeding and breastmilk feeding of infants of mothers who are suspected or confirmed to have COVID-19 in May 2020, which allowed rooming-in, direct breastfeeding with infection protection and control measures and feeding of expressed breastmilk based on mother’s decision after proper counselling [see Additional file 1]. The revised guidelines from Indonesia issued in September 2020 allowed early initiation of breastfeeding, direct breastfeeding with infection control measures and the provision of expressed breastmilk upon family’s preference according to clinical conditions and after receiving education [15]. The Philippines, Thailand and the KK Women’s and Children’s Hospital in Singapore also recommended the provision of donor human milk as a feeding option for infants of mothers with confirmed COVID-19 [13, 14].

### The role of donor human milk banks in a holistic COVID-19 response

For vulnerable infants (e.g. preterm, low birthweight, severely ill) who are unable to breastfeed or access their own mother’s milk, WHO recommends the safe use of donor human milk [16], and reiterated this in the guidance for COVID-19 [9]. When included in a package of interventions to protect, promote, and support breastfeeding, having access to donor human milk can protect the already vulnerable infant from life-threatening complications [17] and help mothers to establish their own milk supply and improve breastfeeding outcomes [18–20]. Human milk banks play a significant role in their function to screen and recruit breastmilk donors, and then to collect, process, screen, store and distribute safe donor human milk [21]. Studies show that the process of Holder pasteurization of donor human milk, which is routinely done in human milk banks, can inactivate the SARS-CoV-2 virus [22, 23].

A total of 35 human milk banks are currently operating in five countries in Southeast Asia (Table 1), with a formal milk bank association convened in only one country (the Philippines). While there is currently insufficient data to accurately assess scale [24], human milk bank services play a critical role in providing donor human milk for vulnerable infants, especially during this crisis. As such, in countries with an established human milk bank, COVID-19 country guidelines should explicitly recommend the use of pasteurized donor human milk as a feeding option when a mother’s own milk is not available. The role of human milk banks in clinical management of COVID-19 needs to be integrated and clearly defined in newborn care guidelines. This is a prerequisite for full alignment with the evidence-based recommendations of WHO.

**Table 1:** Human milk banks in Southeast Asia (as of December 2020)

Country	Facility	City or Province	Recipients of donor human milk
Myanmar	Yangon Central Women’s Hospital	Yangon	Preterm, low birthweight and sick infants in hospital
	Yangon Children’s Hospital		
	Yankin Children’s Hospital		
Philippines	Baguio General Hospital and Medical Center	Baguio City	Preterm, low birthweight and sick infants in hospital; infants in need of donor human milk in humanitarian settings
	Batangas Medical Center	Batangas City	
	Southern Philippines Medical Center	Davao City	
	Western Visayas Regional Medical Center	Iloilo City	
	Ospital ng Malabon	Malabon City	
	Bangkal Health Center	Makati City	
	Makati Medical Center		
	Dr. Jose Fabella Memorial Hospital	Manila	
	Justice Jose Abad Santos General Hospital		
	Ospital ng Maynila Medical Center		
	Philippine General Hospital		
	Bicol Medical Center	Naga City	
	Pasig City General Hospital	Pasig City	
	Rizal Medical Center		
	The Medical City		
	East Avenue Medical Center	Quezon City	
	Philippine Children’s Medical Center		
	Quezon City General Hospital		
	Quirino Memorial Medical Center		
	Jose B. Lingad Memorial Regional Hospital	San Fernando City	
	Eastern Visayas Regional Medical Center	Tacloban City	
	St. Luke’s Medical Center – Global City	Taguig City	
	Taguig District Hospital		
	Cagayan Valley Medical Center	Tuguegarao	
	Zamboanga City Medical Center	Zamboanga City	
Singapore	KK Women’s and Children’s Hospital	Singapore	Preterm, low birthweight and sick infants in hospital; infants in need of donor human milk in the community
Thailand	Faculty of Medicine Siriraj Hospital, Mahidol University	Bangkok	Preterm, low birthweight and sick infants in hospital
	Faculty of Medicine Ramathibodi Hospital, Mahidol University	Bangkok	
	Faculty of Medicine, Chiang Mai University	Chiang Mai Province	
	Faculty of Medicine, Prince of Songkla University	Songkla Province	
Vietnam	Da Nang Hospital for Women and Children	Da Nang	Preterm, low birthweight and sick infants in hospital; infants in need of donor human milk in the community
	Tu Du Maternity Hospital	Ho Chi Minh City	
	Quang Nam General Hospital	Quang Nam Province	

### Challenges and opportunities

Outside of the recommendation on the use of donor human milk in the hierarchy of feeding options, there are currently no WHO guidelines specific to the operation of human milk banks. Countries have thus far relied on existing recommendations from regional milk bank associations [25, 26], expert groups [27], and other countries to inform local standards development. There is also relatively limited guidance regarding donor screening, raw milk handling, collection, and transport of donor human milk in the context of the pandemic, as well as guidance on long-term operational considerations for human milk banks in a post-pandemic setting. While some countries have included disaster risk reduction and response considerations in the operation of their human milk banks prior to the pandemic (e.g. dedicated emergency response supply in the Philippines), human milk banks in the region are generally not designed, nor are currently sufficient in capacity, to respond to large-scale emergencies, especially one as complex as COVID-19. The current pandemic is an opportunity to accelerate the development of WHO guidelines, to advocate for interventions to increase coverage of human milk bank services, or to establish human milk banks in countries where no such services are in place. It is also an opportunity to establish and sustain global partnerships to advance human milk banking through knowledge sharing and joint advocacy. This opportunity has not gone unrecognized. The global human milk bank community recently convened and issued a call to action [24], articulating the need to address fundamental bottlenecks to ensuring safe, equitable, and resilient implementation of human milk bank services in the context of COVID-19 and beyond.

## Conclusions

The aim of this paper is to highlight the need for alignment with WHO guidance on infant feeding in the context of COVID-19, including the need to ensure that human milk bank services are included in the evidence-based package of services for vulnerable infants during the pandemic and beyond.

Given the evolving situation in the region, we, the Regional Human Milk Bank Network for Southeast Asia and Beyond, are a growing network of neonatologists, human milk banking experts, breastfeeding advocates, and infant and young child nutrition experts. We recommend the following to ministries of health, professional associations, and fellow human milk bank practitioners in the region:

1. Revisit and update guidelines that recommend the separation of mothers and infants if the mother has suspected or confirmed COVID-19. The short- and long-term risk of this separation, including disruption of breastfeeding, must be weighed against the potential risk of transmission of COVID-19 using the best available evidence.
2. In the development or updating of national guidelines for obstetric and newborn care in the context of COVID-19:
  - Ensure that the WHO recommended hierarchy of feeding options is followed, with breastfeeding and feeding with mother’s expressed milk prioritized. This can be followed by safe donor human milk from a human milk bank should separation be required or unavoidable (e.g. if the mother is severely ill). Wet nursing may be explored if culturally-acceptable, feasible, and supported by national guidelines [9].
  - Link human milk bank services to clinical management guidelines, and require additional, appropriate safety measures and precautions during raw milk collection, processing, and use (see Additional file 2).
  - Ensure that breastfeeding is protected, promoted, and supported, especially for mothers with suspected or confirmed COVID-19.
3. Adapt human milk bank operations to meet the needs of the current pandemic and to sustain donor human milk supply in the long-term.
4. Contribute to the global learning and research agenda by documenting experiences and lessons learned with human milk banks in the COVID-19 response.
5. Maintain active engagement with regional and global communities of practice such as the Virtual Communication Network of milk bank leaders established in March 2020 [24].

## Supplementary Information

**Additional file 1.** KKHospital Guidance. Guidance on Breastfeeding and Breast Milk Feeding for Suspect and Confirmed COVID-19 - KK Women’s and Children’s Hospital, Singapore. Guidance on Breastfeeding and Breast Milk Feeding for Suspect and Confirmed COVID-19 - KK Women’s and Children’s Hospital, Singapore.

**Additional file 2.** Additional recommendations. Additional recommendations for safe collection and processing of donor human milk during the COVID-19 pandemic. Presents additional measures to enhance safety procedures for donor screening and breastmilk expression to prevent possible contamination of collected donor human milk.

## Data Availability

Not applicable.

## Abbreviations

ASEAN: Association of Southeast Asian Nations
WHO: World Health Organization
