# Supplementary material for: Human milk banks in the response to COVID-19: a statement of the regional human milk bank network for Southeast Asia and beyond
Source: Int Breastfeed J. 2021 Mar 29;16:29. doi: 10.1186/s13006-021-00376-2 (PMC8006108; doi:10.1186/s13006-021-00376-2)
Supplement: Supplementary file 1 — Additional file 1. KKHospital Guidance. Guidance on Breastfeeding and Breast Milk Feeding for Suspect and Confirmed COVID-19 - KK Women’s and Children’s Hospital, Singapore. Guidance on Breastfeeding and Breast Milk Feeding for Suspect and Confirmed COVID-19 - KK Women’s and Children’s Hospital, Singapore. [file 13006_2021_376_MOESM1_ESM.pdf]

Guidance on Breastfeeding and Breast Milk Feeding for Suspect and Confirmed COVID-19

Breastfeeding is the gold standard for infant feeding and nutrition providing optimal health benefits for babies and mothers. An exclusive breast milk diet is recommended for the first 6 months of life. Breast milk provides the best nutrition for optimal growth, contains growth factors, hormones and prebiotics and has enzymes to help with digestion. It is also rich in immune factors which protect the baby from many illnesses.

This guidance is developed based on the evidence available to date about the transmission of COVID-19. According to current evidence, mother-to-baby transmission of SARS-CoV-2 (the causative virus for COVID-19) during pregnancy is unlikely, but the newborn is susceptible to person-to-person transmission after birth.

A small number of babies have been tested positive for the virus after birth. However, it is not known if these babies acquired the virus before or after birth. The virus has to date, not been detected in amniotic fluid, breastmilk or other maternal samples.

The decision for the mother to start breastfeeding or continue breastfeeding should be discussed together with her and her family, bearing in mind the benefits of breastmilk and the recommended precautionary measures to be taken to reduce the risks of transmitting the virus to the child.

As with all confirmed or suspected COVID-19 cases, symptomatic mothers who decides to breastfeed should practice respiratory hygiene and precautionary measures, especially during breastfeeding.

These measures include:

(a) use of a surgical mask.
(b) performing hand hygiene before and after contact with the baby.
(c) routine cleaning and disinfection of high touch surfaces.

Temporary infant separation may be required when a mother has been confirmed to have COVID-19. WHO recommended hierarchy of feeding options should be followed, which include prioritizing on the use of mother's own milk followed by donor human milk from a human milk bank. This is to ensure that breastfeeding is protected, promoted and supported. Pasteurized donor human milk is made available for the vulnerable baby who is unable to breastfeed or without access to his/her own mother's milk.

A shared decision between the parent and the clinical healthcare team should be made on a case-by-case basis, taking into considerations the various factors that may impact the health of the mother and her child.

Samples of breast milk may be sent for COVID-19 tests on Day 1 and Day 3. If the tests are positive, mother will have to cease breastfeeding and stop providing expressed breast milk. All positive tests are to be informed to parents immediately.

SCENARIOS TO CONSIDER (SEE ANNEX 4)

1. COVID-19 positive mother and her newborn OR suspect COVID-19 mother and her newborn

Prior to the birth of the newborn, staff in delivery suite needs to counsel the mother on the benefits of breastfeeding and the precautionary measures that must be taken to minimise the risk of transmission to her newborn if she chooses to breastfeed. For COVID-19 positive mothers, they have higher risk of viral transmission that suspect COVID-19 mothers. The different risk profiles need to be considered during the counselling process.

(a) If mother chooses to room in and breastfeed

A well COVID-19 mother with mild symptoms who has decided to breastfeed her newborn must room-in and practice good respiratory hygiene and precautionary measures (Annex 1). Temporary separation of the newborn from the mother may be necessary if the mother or the newborn is unwell and requires medical care.

(b) If mother chooses not to room in, but will express breastmilk for the newborn

Temporary separation to reduce risk of transmission of COVID-19 to the newborn would be taken if the mother chooses to express her breastmilk for her newborn. The mother is encouraged to express her breast milk regularly to maintain lactation, and her child fed with her expressed breastmilk by the nurse/healthy caregiver. Precautionary measures taken include wearing a surgical mask, washing of hands before and after pumping (Annex 2 and 3).

(c) If mother chooses not to room in and not to express breastmilk OR if counselling is unable to be done prior to delivery

2. Separate mother and baby at birth. Pasteurised donor human milk will be made available for the newborn with no access to their own mother’s milk. **Positive COVID-19 mother and positive COVID-19 newborn.**

If the mother and newborn are not rooming in, to advise mother to room in with the newborn since both the mother and newborn are positive for COVID-19.

Counsel and support mother to breastfeed if she is well and her symptoms are mild. She must continue to practice good respiratory hygiene and precautionary measures (Annex 1) while rooming in with the baby. Temporary separation of the newborn from the mother may be necessary if the mother or the newborn is unwell and requires medical care.

3. Positive COVID-19 mother OR suspect COVID-19 mother with discharged newborn/child

If the mother wants to continue to breastfeed her newborn/child, she should be encouraged to express her breast milk to establish and maintain milk supply. A dedicated breast pump should be provided. Prior to expressing breast milk, mothers should practice hand hygiene. After each pumping session, all parts that come into contact with breast milk should be thoroughly washed and the entire pump should be appropriately disinfected with disinfectant. The expressed breast milk should be stored for feeding to the child by a healthy caregiver back home (refer to Annex 2 and 3).

Ward nurse to discuss with mother to arrange for a friend/relative not under quarantine to collect the bottle of expressed milk from ward. Alternatively, mother may seek assistance via the Stay Home Notice hotline (68125555). Ward nurse to contact the person as provided by the mother on the date and time to collect the EBM with an ice-cooler box with ice-pack.

The EBM will not be stored if mother is unable to make the arrangement for collection. Mother will be advised to pump and discard the milk to maintain lactation.

Annex 1

- Precautionary measures for mother breastfeeding directly

- Wash hands frequently with soap and water or use alcohol-based hand rub, especially before touching baby

- Wear a surgical mask while care and feeding. It is important to:
    - Replace mask as soon as it becomes damp
    - Avoid touching the front of the mask.

- Sneeze or cough into a tissue, immediately dispose of it and use an alcohol-based hand rub or wash hands again with soap and water.

- Regular clean and disinfect the table top surfaces.

Annex 2

Precautionary measures for expressing and storing of breastmilk

- Wear a surgical mask while expressing.

- Wash hands before and after expressing.

- Clean the table top surface before and after pumping with disinfectant wipes.

- A dedicated breast pump is to be provided for use by the mother.

- Clean the outside of the pump with disinfectant wipes, both before and after pumping.

- Use disposable pump kit and storage containers during hospitalisation.

- It is not necessary to wash the breast prior to expressing milk.

- Washing of the breast with soap and water for at least 20 seconds prior to feeding is needed if the mother coughed over her exposed breast or chest.

- Adapt the ‘Bottle Transfer Technique’ and ‘Aseptic Milk Transfer Technique’ by HMBANA to transport the expressed milk to the baby for feeding (Annex 3)

Annex 3

Bottle Transfer Technique and Aseptic Milk Transfer Technique (adapted from HMBANA Apr 14, 2020)

A. **Bottle transfer technique:**
Nurse assisting mother in the room picks up the labelled bottle with expressed breastmilk (EBM) with her gloved hand and place it onto a disposable kidney dish held by a second nurse who is standing outside the room.

B. **Aseptic milk transfer technique**
Prior to receiving the bottle with EBM, the nurse outside the patient's room prepares a clean stainless steel trolley creating a clean and dirty area with the following items:

- Hand Rub and disposable gloves.
- Sterile disposable bottle/s with patient's EBM label.
- Clean plastic bag.
- Disposable kidney dish.

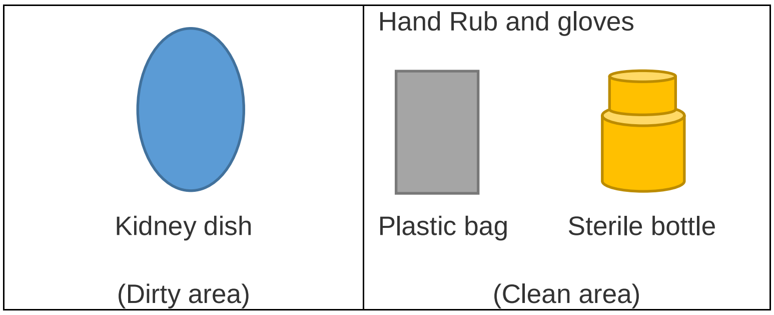

Steps:

1. Don the gloves after hand rub.
2. Open the sterile disposable bottle/s and place the cap/s next to the bottle/s at the clean area.
3. Receive the bottle of expressed breastmilk from the room nurse using the disposable kidney dish.
4. Place kidney dish with the EBM bottle on the trolley at the dirty area.

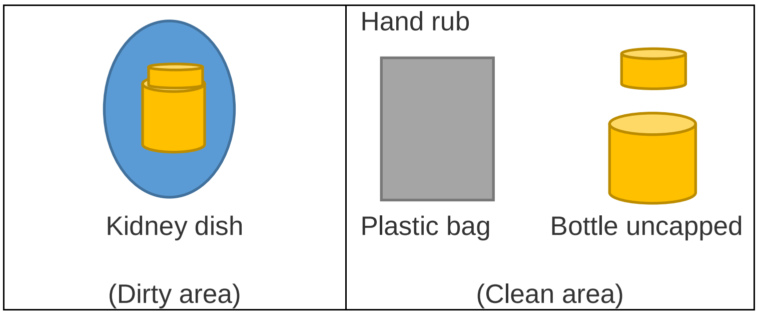

5. Uncap the bottle with EBM and place the bottle-cap onto the kidney dish.
6. Using non-touched, aseptic technique, pour the EBM from the original bottle into the clean labelled sterile bottle on the trolley.
7. Remove and tear the name label from the empty original bottle before disposing with the kidney dish into the biohazard bag.
8. Remove and dispose the gloves and hand rub.
9. Cap the clean bottle with the EBM and place in the individual tray in the fridge or freezer for storage or place in a clean plastic bag and store in the cooler box with ice-pack before activating the porter to send to the baby's location in SCN/NICU immediately.

References:

1. COVID-19 and Breastfeeding. Centres for Disease Control and Prevention. March 18, 2020

2. Care for Breastfeeding Women. Interim Guidance on Breastfeeding and Breast Milk Feeds in the Context of COVID-19. April 28, 2020.

3. COVID-19 and breastfeeding Position paper. World Health Organization Regional office for Europe. April 2, 2020.

4. Shared Decision-Making. Determining a Need for Infant Separation. United States Lactation Consultant Association. April 10, 2020.

5. Frequently Asked Questions: Breastfeeding and COVID-19 For health care workers. World Health Organization. April 28, 2020.

6. Milk Handling for COVID-19 Positive or Suspected Mothers in the Hospital Setting. Human Milk Banking Association of North America. April 14, 2020.

7. Milk Banking and COVID-19. Human Milk Bank Association of North America. www.hmbana.org. April 2, 2020.

Annex 4

Workflow for Counselling of Pregnant Women for Breastfeeding

COVID-19 positive mothers/suspect COVID-19 mothers

Undergo counselling by OG team before delivery

YES

Room in and breastfeed

Mother and newborn in same room.

If suspect mother negative x2 for SARS-CoV-2 PCR, no need to swab newborn.

For newborn with positive mother – to do nasopharyngeal SARS-CoV-2 PCR swab for newborn daily.

Staff to be in full PPE/ googles/ N95 when nursing.

Mother to practise good respiratory hygiene and precautionary measures (Annex 1).

Separation of the newborn from the mother may be necessary if either mother or newborn is unwell and requires medical care.

No room in, for expressed breastmilk

Mother in isolation room.
Baby in SCN** isolation.

If suspect mother negative x2 for SARS-CoV-2 PCR, no need to swab newborn
If mother is positive, to discuss with ID re: swab for baby.

Precautionary measures during expressing of breastmilk as per Annex 2 and 3.

No room in, no breastmilk

Mother in isolation room.
Baby in SCN isolation.

Pasteurised donor human milk will be made available for baby.

*Breast milk will be sent for COVID-109 testing on day 1 and 3
** SCN-special care nursery

NO

Mother in isolation room.
Baby in SCN isolation.

Pasteurised donor human milk will be made available for baby in the interim.

Counsel the mother regarding breastfeeding and rooming after delivery at a suitable time.

If newborn is positive and not rooming in with his/her positive mother:

Advise mother to room in with the newborn since both the mother and newborn are positive for COVID-19.

Encourage breastfeeding.

Mother to practise good respiratory hygiene and precautionary measures (Annex 1).

Temporary separation of the newborn from the mother may be necessary if the mother or the newborn is unwell and requires medical care.

Updated 11 May 2020
