# Supplementary material for: Human milk banks in the response to COVID-19: a statement of the regional human milk bank network for Southeast Asia and beyond
Source: Int Breastfeed J. 2021 Mar 29;16:29. doi: 10.1186/s13006-021-00376-2 (PMC8006108; doi:10.1186/s13006-021-00376-2)
Supplement: Supplementary file 2 — Additional file 2. Additional recommendations. Additional recommendations for safe collection and processing of donor human milk during the COVID-19 pandemic. Presents additional measures to enhance safety procedures for donor screening and breastmilk expression to prevent possible contamination of collected donor human milk. [file 13006_2021_376_MOESM2_ESM.docx]

**Additional recommendations for safe collection and processing of donor human milk during the COVID-19 pandemic**

Routine safety and quality assurance measures during donor screening and serologic tests, raw milk collection, pasteurization and microbiological testing should be strictly implemented to ensure donor milk is free of any contamination before it is dispensed to recipients.

With the current COVID-19 pandemic, consider the following additional measures to strengthen safety procedures in the handling and processing of donor human milk:

1. **Donor screenings**
2. Should include information on travel to or residence in a country/area with reported local transmission of COVID-19, history of recent illness, including among family members and close contacts, with emphasis on the following signs and symptoms: fever (temperature of more than 380C), cough, difficulty of breathing, sore throat, nasal congestion, diarrhea, cluster of influenza-like illness in the household, community of residence, or workplace in the past 14 days.
3. Other pertinent information required by national guidelines for COVID-19 case identification

**2. Breastmilk expression**

Donor mothers who reside in areas where COVID-19 is spreading in the community must wear a face mask when expressing their milk. They should wash their hands thoroughly with soap and water before and after expressing. If expressing breastmilk using a pump, they should be advised to wash their hands before touching any pump components or containers. The recommended procedures for proper pump cleaning and disinfection should be followed (wash the pump and containers after every use with liquid soap such as dishwashing liquid and warm water, then rinse with hot water for 10-15 seconds) (WHO, 2020a).
